# A novel magnetic orthodontic bracket system for static magnetic field generation: a finite element analysis

**DOI:** 10.3389/fdmed.2026.1962429

**Published:** 2026-10-14

**Authors:** Reuben Bensy Thomas, Vrinda Vijayan M, Achal Balachandran, Shouvik Dam, Naveen Pulikkottil Sunny, M. S. Ravi

**Affiliations:** Department of Orthodontics and Dentofacial Orthopaedics, Nitte (Deemed to be University), AB Shetty Memorial Institute of Dental Sciences (ABSMIDS), Mangalore, Karnataka, India

**Keywords:** accelerated orthodontics, finite element method, magnetic field, magnetism, orthodontic brackets

## Abstract

**Introduction:**

Magnetic forces have the potential to accelerate orthodontic tooth movement. Miniature sized neodymium-iron-boron (Nd–Fe–B) magnets along with biocompatible coating materials are now available enabling a re-evaluation of magnetic usage in orthodontic brackets. The study was done to assess the viability of embedding magnetic material into orthodontic brackets for generating localized static magnetic fields.

**Methods:**

An intra-oral scan of a patient with orthodontic brackets was obtained and reconstructed into a three-dimensional dentition model, and exported in STL format. Neodymium–iron–boron (Nd–Fe–B) magnets and soft magnetic iron yokes were designed and positioned on the model. The magnetic elements were placed within the bracket body between the bracket base and archwire slot in an alternating magnet–yoke arrangement, such that one bracket contained only the magnet and the adjacent bracket contained the soft iron yoke. Magnetostatic finite element analysis was performed using Gmsh and GetDP to evaluate magnetic flux density distribution and field propagation.

**Results:**

Finite element simulation displayed creation of localized magnetic field, with a peak magnetic flux density of about 0.72–0.83 Tesla immediately adjacent to the magnetic elements (0.72 T under the primary, locally-oriented magnetization configuration; up to 0.83 T under the supplementary global-axis configuration), decaying by two to three orders of magnitude within a few millimetres; the far-field inter-bracket field values are mesh-sensitive and are therefore reported as provisional.

**Conclusion:**

This study demonstrated the feasibility of establishing a localized static magnetic field around the embedded brackets, with simulated field strength decaying by two to three orders of magnitude within a few millimetres of the bracket surface. This approach may hold potential for influencing biological processes relevant to orthodontic tooth movement. However, its clinical significance requires validation through further clinical studies.

## Introduction

Orthodontic tooth movement depends on co-ordinated bone remodelling, which is influenced by local cellular activity (1). Experimental studies have suggested that static magnetic fields may modulate the behaviour of osteoblasts and osteoclasts, regulating bone formation and resorption, suggesting they are implicated in bone remodelling (2–4).

However, despite these observations, there is currently no orthodontic appliance capable of delivering a localized and sustained static magnetic field directly to the periodontal tissues throughout treatment.

Magnetism is a fundamental physical phenomenon induced by the movement of electric charges and the spin of electrons, which gives rise to magnetic fields and dipole interactions (5). Biological systems are sensitive to static magnetic fields (SMFs) and their effects on cell growth, differentiation, and metabolism have been identified (6, 7). As orthodontic tooth movement requires controlled bone alteration in the periodontal ligament and alveolar bone, magnetic field effects may attract interest in orthodontics. Traditionally, magnetism in orthodontics was explored as a source of mechanical force, as seen in earlier magnetic functional appliances to modulate mandibular position and growth (8). Magnetic systems have also been used to facilitate midline diastema closure, using attractive forces to approximate teeth, and canine retraction with magnetic repulsion or attraction as an alternative to elastics and coil springs (9, 10). However, clinical application was hindered by several limitations, including the bulkiness of magnets, difficulty in force control, corrosion issues and patient discomfort. The development of high-strength rare-earth magnets like neodymium-iron-boron (Nd–Fe–B) has made possible miniaturisation without significant sacrifice of magnetic field intensity (11). These magnets are now readily available at millimetre scale, fitting within the confines of orthodontic brackets. Crucially, biocompatible coating materials such as parylene have been developed to encapsulate magnets, preventing direct contact with the oral environment and the highly corrosive conditions found there, to which Nd–Fe–B magnets are susceptible (12). Moreover, embedding the magnet within the bracket offers mechanical advantages i.e., it prevents movement, displacement or ingestion, avoids interference with soft oral tissues, and allows it to passively occupy the intended space without compromising orthodontic mechanics like archwire attachment. An orthodontic bracket with a built-in magnetic system would therefore integrate a potential novel system without disruption to established mechanics. The concept of this study involves integrating Nd–Fe–B magnets with alternating soft magnetic iron yokes with orthodontic brackets along the arch to create localized static magnetic fields. Studies have demonstrated the potential of static magnetic fields to modify cellular behaviour and bone remodelling, thereby enhancing the rate of orthodontic tooth movement (13, 14). The proposed concept seeks to leverage this as a biological adjunct rather than a direct substitute for orthodontic mechanical forces, offering to potentially generate a biologically useful magnetic field while maintaining its mechanical functions.

The primary objective of this study was to determine whether static magnetic fields generated by Nd–Fe–B magnets embedded within orthodontic brackets could reach a magnitude, at clinically relevant tissue depths, sufficient either to generate a direct mechanical force contributing to orthodontic tooth movement, or to fall within the range of field intensities reported to influence periodontal and osseous cellular activity in prior *in vitro* and *in vivo* studies.

## Methodology

This study was approved by the Institutional Ethics Committee of Nitte (Deemed to be University), A.B. Shetty Memorial Institute of Dental Sciences, Mangalore, India (Reference No. ETHICS/ABSMIDS/722/2026). Informed consent was obtained from the patient for the use of anonymized Intra-oral scan for research purposes.

A patent application for the proposed concept has been published (Reference No. 202641043772 A), and the application is currently pending grant.

The FEM simulation was performed on orthodontic brackets incorporating magnetic components, arranged alternately such that a magnet was integrated into one bracket and a yoke into the adjacent bracket (Figures 1, 2).

**Figure 1:**
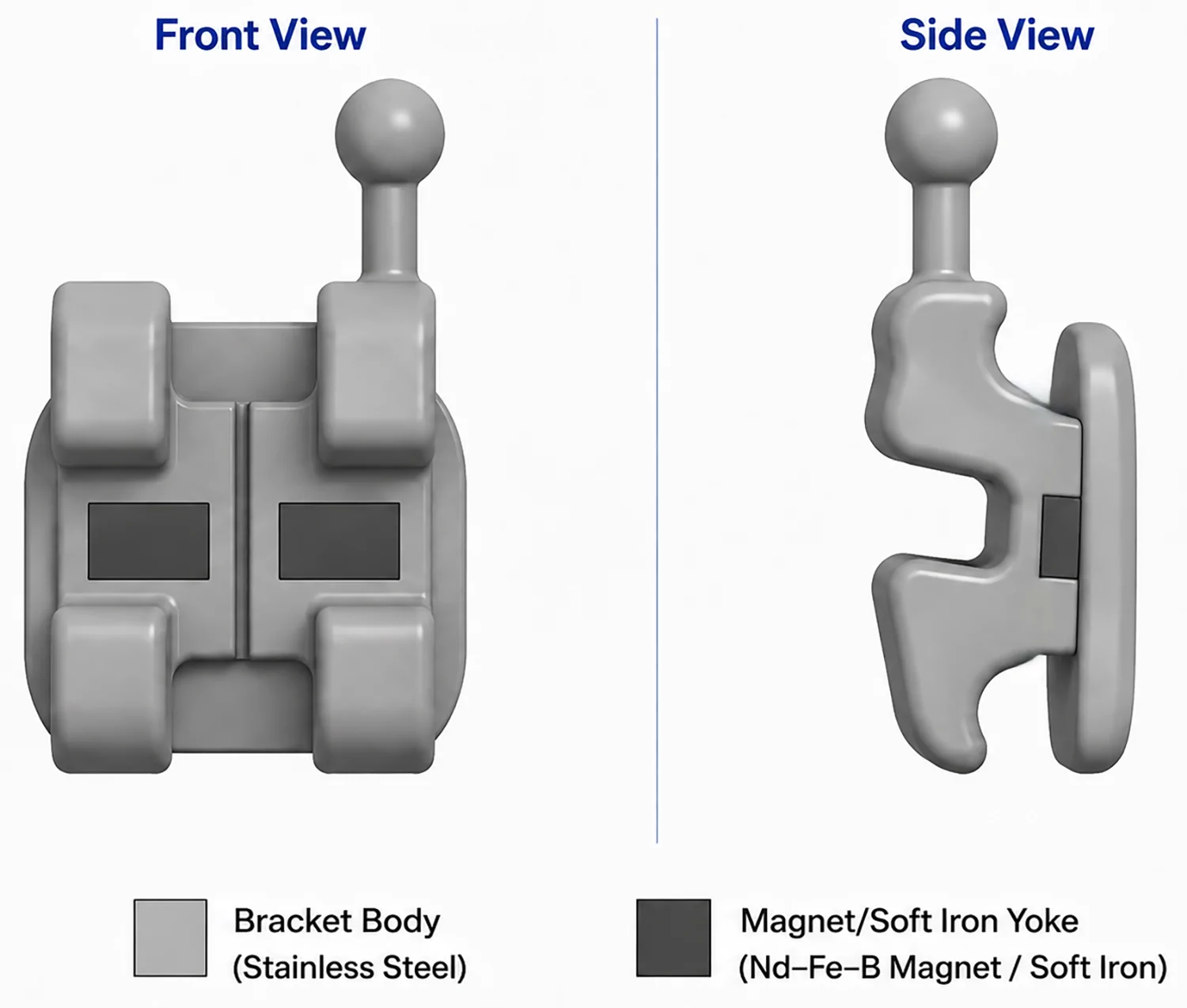
Front and lateral view of the proposed bracket. Magnet or Soft iron yoke are encapsulated within the body of the bracket.

**Figure 2:**
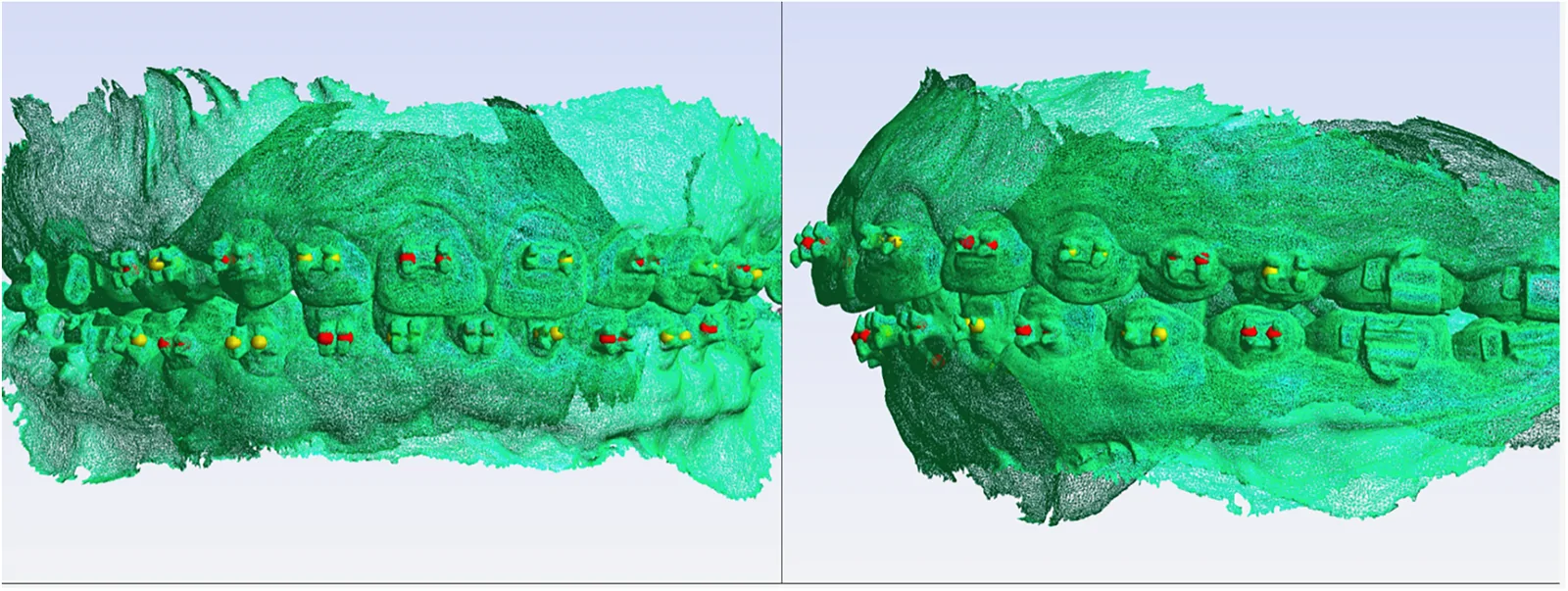
Front and side view of the alternate magnet-yoke arrangement in brackets (red volumes depicting Nd–Fe–B magnets and yellow depicting iron yokes).

Because the primary objective of this study was to establish whether embedding Nd–Fe–B magnets within orthodontic brackets can generate a localized static magnetic field of biologically relevant intensity, and to characterize its spatial decay, rather than to model patient-specific periodontal or osseous anatomy, an intra-oral scan was considered a sufficient and appropriate source of anatomical input for this study: it provided anatomically realistic bracket dimensions, inter-bracket spacing, and arch curvature consistent with clinical practice. Intra-oral scans of a patient with orthodontic brackets were exported as a Stereolithography (.stl) file and imported into Gmsh version 4.15.2 (15). Because the scan was acquired as a surface (non-manifold) mesh, it could not be converted into a closed solid volume; it was therefore used to determine anatomically realistic bracket positions and was not itself assigned distinct magnetic material properties. Modifications were made to the bracket area for embedding magnetic materials. Cuboidal void areas were created within the orthodontic bracket between the archwire slot and bracket base on mesial and distal aspects of the brackets, which would be occupied by magnetic material. Additional cuboidal volumes were introduced to fill these void spaces and the areas were assigned as neodymium-iron-boron (Nd–Fe–B) magnets and soft magnetic iron yokes. The bracket body itself was not separately meshed as a distinct solid region; the space it occupies was instead treated as part of the surrounding ambient (air-equivalent) domain in the solved model. This simplification is supported by the magnetic properties of the bracket material: austenitic stainless steel, the standard orthodontic bracket alloy, has a relative permeability of approximately 1.05, only marginally higher than that of air (μr = 1). This stands in contrast to the soft iron yoke, whose permeability (μr = 2,000) is more than three orders of magnitude higher than air and is the dominant driver of local flux concentration in this model. The yoke's permeability governed the simulated field pattern, while treating the bracket body as air-equivalent introduced a comparatively minor perturbation, consistent with this study's objective of characterizing field generation and decay rather than fine-grained field distribution within the bracket body itself.

Material properties, boundary conditions, and solver settings used in the simulation are summarized in Table 1.

**Table 1:** Consolidated material properties, boundary conditions, and solver settings used in the finite element model.

Region/Parameter	Symbol	Value	Basis
Air domain	μr	1	Standard (vacuum-equivalent)
Physical bracket material (not separately modeled)	μr	1.05	Physical property of austenitic stainless steel; included for air-equivalent modelling approximation
Soft magnetic iron (yoke)	μr	2,000	Consistent with semi-processed low-carbon steel (Fe–0.04%–0.06%C), reported permeability ≈2,000
Nd–Fe–B (magnet)	μr	1.05	Matches recoil permeability of sintered Nd–Fe–B magnets across grades, per manufacturer datasheet
Nd–Fe–B remanence	Br	1.2 T	Falls within the N35AH grade range (Br = 1.17–1.22 T), consistent with commonly used sintered Nd–Fe–B magnet grades
Magnet/yoke element size	—	0.8 mm cuboid (edge)	Design specification (this study)
Outer boundary condition	—	Magnetic insulation	Standard open-boundary approximation
Solver convergence tolerance	—	1 × 10⁻⁶	Solver setting (this study)
Mesh (medium, used for all runs)	—	2,897 nodes; 16,534 tetrahedral elements	Mesh convergence analysis (Table 3)

The magnetic material and the yoke were placed in alternating positions along the dental arch, with the intent of directing magnetic flux across inter-bracket spacing through the iron yokes adjacent to the magnets. Two magnetization configurations were modelled: a primary, locally-oriented (arch-normal) configuration, in which each magnet's magnetization follows the local bracket orientation, and a supplementary configuration using a single fixed global Z-axis vector (see results). A surrounding air volume encompassing the bracket and dentition was defined for the analysis. All geometry was then discretized into tetrahedral finite elements and processed.

A magnetic vector potential formulation was used in GetDP to compute the static magnetic field distribution.

Mesh convergence analysis was performed to ensure numerical stability and solution independence from mesh density. Three progressively refined tetrahedral meshes were generated and the peak magnetic flux density values were compared.

Post-processing and visualization of magnetic field distributions were performed within the Gmsh environment. Magnetic flux density values were assessed at varying inter-bracket distances to evaluate spatial attenuation characteristics.

## Results

Magnetostatic finite element simulations demonstrated successful generation of localized static magnetic fields around the embedded Nd–Fe–B magnetic components incorporated within the orthodontic brackets. Regions of maximum magnetic flux density were concentrated immediately adjacent to the magnetic elements, with a peak value of approximately 0.72 T under the primary (locally-oriented) configuration and 0.80–0.83 T under the supplementary global Z-axis configuration. The highest flux densities were localized at the magnet–yoke interfaces, consistent with local flux concentration arising from the permeability contrast between the magnet and yoke materials at their shared interface, independent of the arch-wide flux-direction.

As the distance from the magnetic components increased, a progressive reduction in magnetic flux density was observed, demonstrating spatial attenuation of the magnetic field and confinement of the strongest magnetic effects to the vicinity of the brackets. Field concentration at each magnet-yoke interface was observed along the arch, though this reflects local permeability contrast rather than a directed inter-bracket flux path. Distinct zones of elevated magnetic flux density were observed around each magnetic component along the dental arch, while lower field intensities were present within the surrounding air domain and non-magnetic regions. Embedded magnetic elements were capable of establishing localized magnetic field environments without generating widespread field distribution throughout the entire model (Figure 3).

**Figure 3:**
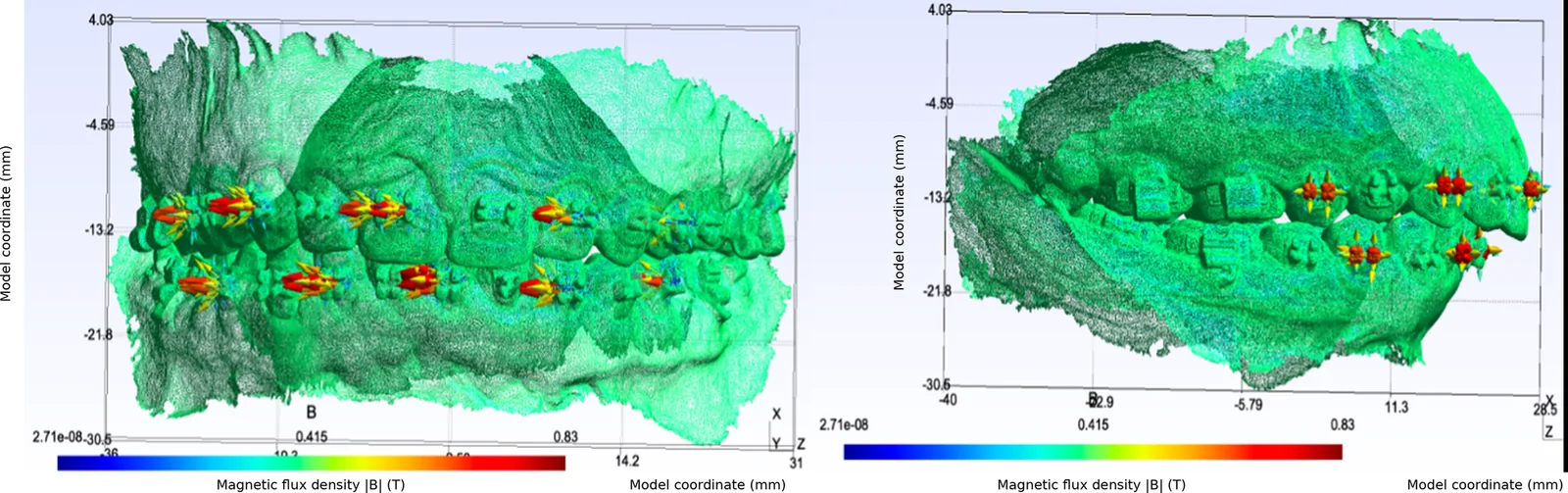
Magnetostatic simulation solved using GetDP (global Z-axis magnetization configuration; retained as a supplementary comparison — see Figure 5 for the primary, locally-oriented configuration adopted in this study).

Vector field analysis of the supplementary global Z-axis configuration further demonstrated the directional characteristics of magnetic flux propagation. Magnetic flux vectors originated from the Nd–Fe–B magnetic elements and were directed toward the adjacent soft iron yokes, forming localized magnetic pathways across the inter-bracket regions. Regions represented by red and orange vectors corresponded to areas of higher magnetic flux density, whereas smaller blue vectors indicated progressive attenuation of the magnetic field with increasing distance from the magnetic assemblies. All flux vectors point in the same nominal direction rather than alternating with local bracket orientation around the arch; within that constraint, localized static magnetic fields were nonetheless established between adjacent brackets (Figure 4).

**Figure 4:**
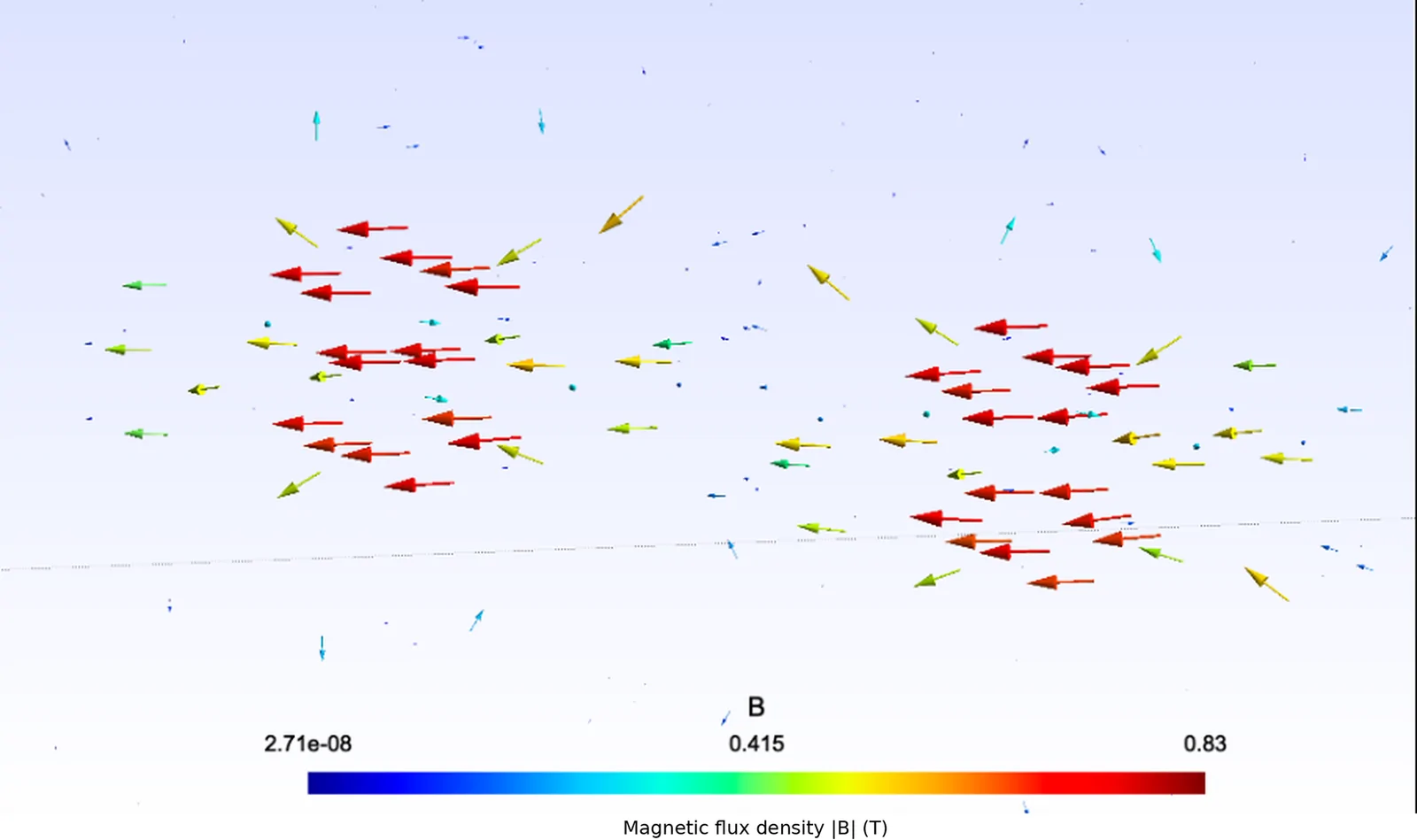
Vector plot obtained from finite element magnetostatic simulation showing the direction and relative magnitude of magnetic flux density (B-field) generated by the alternating Nd–Fe–B magnet and soft iron yoke bracket configuration. Red and orange vectors indicate regions of higher magnetic flux density concentrated around the embedded magnetic assemblies, while smaller blue vectors represent attenuation of the magnetic field within the surrounding air domain (global Z-axis magnetization configuration; retained as a supplementary comparison — see Figure 5 for the primary, locally-oriented configuration adopted in this study).

The uniform orientation of flux vectors visible arises directly from the model specification, in which magnetization was defined as a single fixed vector along the global Z-axis and applied identically to every magnet element, rather than a direction following each bracket's local orientation around the arch curvature, as a modelling simplification.

To quantify the impact of this simplification, a comparative analysis was performed in which each of the twenty magnet elements was instead assigned a magnetization direction oriented radially outward from a best-fit arch centerline, rather than the single global Z-axis vector, with mesh, materials, and solver settings otherwise identical. At the twenty inter-bracket sampling sites used elsewhere in this study, the locally-oriented model produced a mean magnitude change of −34% relative to the global-axis model (individual sites ranging from −66% to +31%) and, more substantially, a mean field-direction change of approximately 95° (range 2°–179°), with several sites showing a near-complete reversal of field orientation (Figure 5).

**Figure 5:**
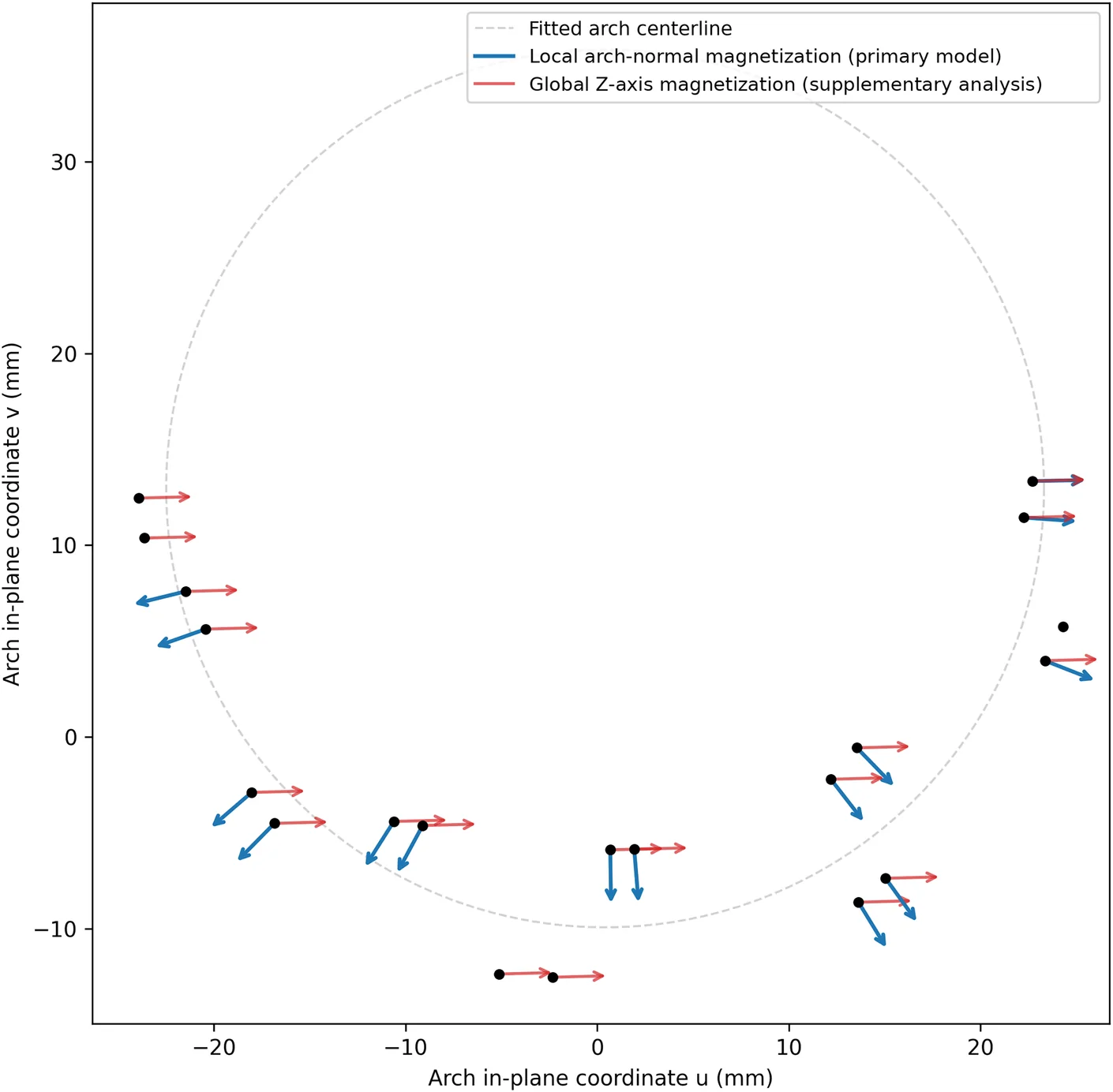
Comparison of the global Z-axis magnetization assumption (red arrows; supplementary comparison) against the locally-oriented, arch-normal magnetization direction (blue arrows) adopted as the primary model in this study, derived from a best-fit arch centerline, for each of the twenty magnet elements, projected into the best-fit arch plane. The two assumptions coincide only at one end of the arch (site 20); elsewhere the global-axis assumption diverges substantially from the anatomically appropriate local direction.

Table 2, the inter-bracket field value, and the adhesive-layer field estimates below use the locally-oriented (arch-normal) magnetization configuration.

**Table 2:** Simulated magnetic flux density as a function of radial distance from the magnet surface, averaged across all twenty magnet-containing bracket sites (*n* = 20; range given as min–max across sites), computed using the locally-oriented (arch-normal) magnetization configuration adopted as the primary model (see results).

Distance from magnet surface (mm)	Mean |B| (T)	Min |B| (T)	Max |B| (T)
0.0	0.6300	0.0765	0.6980
1.0	0.0364	0.0079	0.1230
2.0	0.0113	0.0025	0.0544
3.0	0.0043	0.0013	0.0071
5.0	0.0011	0.0003	0.0026
10.0	0.0002	0.0001	0.0006

To characterize radial field attenuation from the individual magnet surfaces, magnetic flux density was sampled at predefined radial distances and averaged across all twenty magnet-containing bracket sites (Table 2).

The 0.0 mm row of Table 2 (mean 0.630 T, range 0.077–0.698 T across sites) is lower than the peak values reported above because it represents the radial field at the magnet surface averaged across sites, rather than the locally concentrated flux at the magnet-yoke interface (see discussion).

Separately, in the full-arch model, the magnetic flux density at the midpoint between the nearest magnet and yoke elements (3.93 mm, centre-to-centre) was 0.0207 T under the primary (locally-oriented) magnetization configuration, compared with 0.061 T under the supplementary global Z-axis configuration at the same location. The simulated field showed marked attenuation with increasing distance from the magnetic components, with reductions of approximately two to three orders of magnitude over the sampled distance range.

Mesh convergence analysis, performed for the supplementary global Z-axis magnetization configuration, demonstrated numerical stability of the finite element model. The coarse mesh (element size 0.5 mm) yielded a maximum magnetic flux density of 0.74 T, while the medium (0.25 mm) and fine (0.1 mm) meshes produced values of 0.81 T and 0.82 T, respectively. The difference between the medium and fine mesh solutions was less than 2%, indicating convergence of the peak magnetic flux density with mesh refinement. Based on this result, the medium mesh configuration was selected for subsequent simulations as it provided an appropriate balance between computational efficiency and numerical resolution (Table 3).

**Table 3:** Mesh convergence analysis (supplementary global Z-axis magnetization configuration).

Mesh Type	Element Size	Maximum Magnetic Flux Density
Coarse	0.5 mm	0.74 T
Medium	0.25 mm	0.81 T
Fine	0.1 mm	0.82 T

An additional convergence check was performed at the far-field inter-bracket sampling sites used to characterize the arch-wide field distribution. The medium mesh (16,534 elements) was uniformly refined twice, to 132,272 and 1,058,176 tetrahedral elements respectively, and the inter-bracket field was re-sampled at all twenty sites at each mesh density (Table 4). The mean inter-bracket field changed from 8.55 mT (medium) to 6.20 mT (8× refinement) to 5.36 mT (64× refinement); individual sites shifted by as much as 54% between the first two mesh densities and by as much as 40% between the second and third, with the step size roughly halving at each refinement.

**Table 4:** Far-field (inter-bracket) mesh convergence analysis at twenty arch sampling sites, supplementary to the near-magnet convergence check in Table 3.

Mesh density	Elements	Mean inter-bracket B	Max single-site change from previous level
Medium (baseline, used for all other analyses)	16,534	8.55 mT	—
8× refined	132,272	6.20 mT	54%
64× refined	1,058,176	5.36 mT	40%

The baseline (medium) mesh comprised 2,897 nodes and 16,534 tetrahedral elements distributed across the magnet, yoke, and surrounding air domains. An internal consistency check was performed by comparing the simulated on-axis magnetic flux density from an isolated 0.8 mm Nd–Fe–B magnet element with the corresponding closed-form analytical solution for a uniformly magnetized rectangular prism magnet (16). Simulated and analytical values agreed within the same order of magnitude across the tested range (0.4–5 mm from the magnet surface), with near-exact agreement at 1 mm separation.

Sensitivity of the far-field results to two further methodological factors was also examined. Since the original mesh geometry could not be regenerated with a larger air domain, the sensitivity of the inter-bracket field to the treatment of the existing outer domain boundary was tested by comparing the default (open) boundary condition against an explicit constraint enforcing zero tangential vector potential (a “closed” boundary) on the outer mesh surface; the mean inter-bracket field changed by only 0.54% (maximum 2.87% at any single site). A one-at-a-time material-property sensitivity analysis at the inter-bracket sites showed the field scales linearly with magnet remanence (an 8% change in Br producing an 8.3% change in field, consistent with the linear magnetostatic formulation used here), a modest sensitivity to magnet permeability (2%–4% over a realistic range), and negligible sensitivity to yoke permeability (under 0.1% even across a fourfold range) (Table 5).

**Table 5:** Sensitivity of the inter-bracket field to outer domain boundary treatment and material properties, mean across twenty arch sites.

Factor varied	Condition	Mean inter-bracket B	% change vs. baseline
Outer boundary condition	Open (baseline/default)	8.55 mT	—
Outer boundary condition	Closed (zero tangential A)	8.60 mT	+0.54% (max 2.87% at any site)
Magnet remanence Br	1.10 T (−8%)	7.84 mT	−8.3%
Magnet remanence Br	1.30 T (+8%)	9.26 mT	+8.3%
Magnet permeability μr	1.00	8.35 mT	−2.3%
Magnet permeability μr	1.15	8.92 mT	+4.3%
Yoke permeability μr	1,000 (−50%)	8.55 mT	∼0.0%
Yoke permeability μr	4,000 (+100%)	8.55 mT	∼0.0%

Sensitivity analysis was performed to evaluate how the predicted inter-bracket field, and the resulting Maxwell-stress force estimate, respond to key model parameters. As the bracket geometry does not permit parametric resizing without manual reconstruction of each bracket, this analysis used a simplified idealized unit-cell model: a single 0.8 mm Nd–Fe–B magnet cube and a single 0.8 mm soft iron yoke cube, facing one another across an 8 mm gap (the midpoint of the 7–9 mm clinically relevant inter-bracket spacing used elsewhere in this study), embedded in a large air domain, using the same magnetostatic formulation and confirmed material properties (μr = 2,000 for the yoke) as the full model. Baseline parameters were then varied one at a time: magnet remanence (representing N42 and N52 grade Nd–Fe–B magnets, in addition to the baseline 1.20 T value), magnet cube edge length (0.6 and 1.0 mm), yoke cube edge length (0.6 and 1.0 mm), and inter-bracket distance (7 and 9 mm).

Inter-bracket distance and magnet size had the largest influence on the force estimate, with changes of up to +163.8% (1.0 mm magnet) and −79.6% (0.6 mm magnet) relative to baseline, and up to +45.3% (7 mm gap) and −64.8% (9 mm gap). Magnet remanence had a smaller, monotonic effect (+17.4% to +42.0% across the N42–N52 grade range), and yoke size had the smallest effect of the four parameters tested (−14.4% to +10.2%).

For the direct mechanical interaction between a single neighbouring magnet -yoke pair, the baseline force estimate was 2.67 × 10⁻⁷ N, with a maximum of 7.03 × 10⁻⁷ N across the tested parameter range (Table 6).

**Table 6:** One-at-a-time sensitivity analysis: effect of magnet remanence, magnet and yoke dimensions, and inter-bracket distance on the simulated gap-midpoint field and Maxwell-stress force estimate, using the idealized single magnet-yoke unit-cell model.

Parameter varied	Value	B at gap (T)	Force estimate (*N*)	% change vs. baseline
Baseline	0.8 mm magnet, 0.8 mm yoke, 8 mm gap, Br = 1.20T	0.00082	2.67 × 10⁻⁷	—
Remanence (Br)	1.30 T (N42 grade)	0.00089	3.13 × 10⁻⁷	+17.4%
Remanence (Br)	1.43 T (N52 grade)	0.00098	3.79 × 10⁻⁷	+42.0%
Magnet size	0.6 mm cube	0.00037	5.45 × 10⁻⁸	−79.6%
Magnet size	1.0 mm cube	0.00133	7.03 × 10⁻⁷	+163.8%
Yoke size	0.6 mm cube	0.00086	2.94 × 10⁻⁷	+10.2%
Yoke size	1.0 mm cube	0.00076	2.28 × 10⁻⁷	−14.4%
Inter-bracket distance	7 mm	0.00099	3.87 × 10⁻⁷	+45.3%
Inter-bracket distance	9 mm	0.00049	9.37 × 10⁻⁸	−64.8%

The influence of the stainless-steel orthodontic archwire on the simulated field was also assessed. The archwire's effect was approximated by reclassifying the mesh air elements lying within 1.2 mm of a fitted arch-shaped wire path as a separate material region. Under a magnetic permeability representative of nickel-titanium or austenitic stainless-steel archwire (μr ≈ 1.02, both essentially non-magnetic), the inter-bracket field changed by less than 0.5% at any sampled site. Under a conservative upper-bound permeability (μr = 100, well above any wire alloy used clinically), most sites changed only modestly (mean −2.6%), though localized changes of up to 53% were observed near the ends of the modelled wire span.

The magnetic flux density at the estimated adhesive/cement bonding layer (Space between each magnet bracket's base and the tooth surface, offset inward by a clinically typical bond thickness of 0.1–0.3 mm) was also extracted from the primary (locally-oriented) model solution. Across the twenty magnet-containing bracket sites, this ranged from approximately 28 to 680 mT depending on bracket position and assumed bond thickness (mean 336 mT at 0.15 mm) (Table 7).

**Table 7:** Summary of the archwire influence and adhesive/cement bonding-layer field analyses.

Analysis	Condition	Result
Archwire influence	μr = 1.02 (realistic: NiTi/austenitic stainless steel)	<0.5% change at any of 20 sites
Archwire influence	μr = 100 (conservative worst case)	Mean −2.6%; localized changes up to 53% near modelled wire ends (likely a finite-segment artefact)
Adhesive-layer field (0.15 mm bond thickness)	Mean across 20 magnet brackets	336 mT (range 28.6–668.6 mT)
Adhesive-layer field vs. assumed bond thickness	0.10/0.15/0.20/0.30 mm	486.9/336.1/241.9/143.4 mT (mean)

## Discussion

This study explored the viability of incorporating embedded Nd–Fe–B magnetic components into a standard orthodontic bracket system to deliver localized static magnetic fields. Finite element simulations yielded peak magnetic flux densities of approximately 0.72–0.83 Tesla immediately adjacent to the magnets (0.72 T under the primary, locally-oriented magnetization configuration; up to 0.83 T under the supplementary global-axis configuration), concentrated specifically at the magnet–yoke interfaces. Table 2 complements this peak value with a separate, deliberately-chosen metric: the radial decay of the field outward from each magnet surface, away from the arch, characterizing how the field diminishes into the surrounding tissue space rather than the locally concentrated flux at the magnet–yoke interface itself. The two therefore capture different aspects of the same field by design (the peak establishes the upper bound of field intensity at the design's most concentrated point, while the radial profile characterizes its attenuation into the region of actual biological interest). The inter-bracket field reflects contributions from the whole magnet ensemble rather than the nearest pair alone, and small rare-earth magnetic elements are capable of creating strong localized fields even within the limited space available inside an orthodontic bracket.

A central methodological question concerns the direction assumed for each magnet's eas*y* axis. The primary model reported here assigns each magnet a locally-oriented (arch-normal) magnetization, in place of a single global Z-axis vector; the global-axis configuration is retained only as a supplementary sensitivity comparison (Figure 5). At the twenty inter-bracket sampling sites used throughout this study, the locally-oriented model produced a mean magnitude change of −34% relative to the global-axis model and a mean field-direction change of approximately 95°, demonstrating that magnetization direction is a first-order determinant of the inter-bracket field across the arch — varying with the tooth-specific arch-normal orientation at most positions, and coinciding with the global-Z assumption only at the one location where the two happen to align. This sensitivity is precisely why we tested it directly rather than relying on a single global convention, and on that basis the locally-oriented configuration was adopted as the primary, clinically-representative model for all arch-wide results reported here, including Table 2, the inter-bracket field value, and the adhesive-layer field estimates below. Two quantities are essentially unaffected by this choice. The near-magnet peak flux density (Table 3) changes only modestly (0.72 T under the local configuration versus 0.80–0.83 T under the global configuration, at the same mesh density), because it is dominated by each magnet's own local self-magnetization rather than its orientation relative to the rest of the arch; the mesh-convergence demonstration in Table 3 therefore remains valid as a numerical check regardless of which configuration is treated as primary. The Maxwell-stress force estimate (Table 6) is likewise unaffected, since it is computed from an isolated magnet–yoke pair whose local facing geometry, not an arch-wide coordinate convention, defines the interaction and therefore does not depend on this choice.

This same unit-cell model was used, in preference to the full-arch simulation, to estimate the direct mechanical interaction between a single neighbouring magnet–yoke pair (17), because it isolates the local interaction from contributions arising from the remaining magnetic elements in the full-arch model. Across the tested parameter range, the estimated force remained on the order of 10⁻⁸–10⁻⁷ N, substantially below conventional orthodontic force levels, and decreased rapidly with increasing separation, consistent with the strong distance dependence of magnetic-field interactions (18). This conclusion was robust across the full sensitivity analysis (Table 6): even the highest force estimate obtained (7.03 × 10⁻⁷ N, for a 1.0 mm magnet) remained several orders of magnitude below typical orthodontic force levels, and orthodontic forces effective in tooth movement are considerably stronger regardless of movement type, making a system based on direct mechanical tooth movement via magnetic force seem unlikely. The absolute field values from this idealized unit-cell model are substantially lower than the inter-bracket field reported from the full-arch model — 0.0207 T at 3.93 mm separation under the primary (locally-oriented) configuration, or 0.061 T under the supplementary global-axis configuration at the same location. Part of this gap reflects the uniform global-axis magnetization in the supplementary configuration, where all twenty magnet elements around the arch are magnetized in the same direction and their fields superpose additively rather than partially cancelling; the primary configuration reduces but does not eliminate the gap, since the full-arch inter-bracket field still reflects contributions from the wider magnet ensemble rather than the isolated pair modelled here. This full-arch value is therefore best interpreted as a measure of the simulated inter-bracket magnetic field rather than as a direct estimate of the local force between a single magnet and yoke.

While no strong mechanical forces were detected between adjacent brackets, localized static magnetic fields were present around the embedded magnetic components and extended into the surrounding model domain; these fields may be of greater therapeutic interest than the mechanical pathway. Static magnetic fields have been reported to affect osteoblast proliferation and activity, cellular division, angiogenesis, and remodelling (19–23), and since periodontal ligament and alveolar bone remodelling are critical to orthodontic tooth movement, the localized fields generated here could plausibly influence tooth movement through changes in the surrounding tissue; the proposed bracket system could in this sense serve as a delivery vehicle for localized static magnetic fields. It is useful to compare the simulated decay values against the field strengths actually used in the cited studies. Shan et al. reported increased osteoclast activity and altered tooth movement in mice across an applied field range of approximately 20–204 mT (13); Luo et al. used a local exposure of 200 ± 20 mT at the buccal mucosa adjacent to the target tooth in rats, comparable to the 200 ± 50 mT surface strength of the clinical appliance their model approximated, and observed a significant increase in tooth movement distance (14); and Chiu et al. used continuous exposure to a 0.4 T field to promote osteoblast-like cell differentiation *in vitro* (19). Taken together, these studies used field strengths of approximately 20–400 mT. The present simulation, under the primary locally-oriented configuration, predicts a comparable field magnitude of approximately 11–36 mT within roughly 1–2 mm of the magnet surface (Table 2), falling to single-digit mT and below by 3 mm and beyond — suggesting that a biologically relevant field magnitude may only be plausible very close to the bracket itself, rather than at the depth of the periodontal ligament or alveolar bone crest, in most clinical scenarios. This comparison should be read cautiously: the cited studies used whole-appliance or directly-applied local exposure in different species or *in vitro* culture, with different field geometries and durations than the embedded magnet–yoke configuration modelled here, and is therefore offered as a plausibility check rather than a direct validation.

Two further interactions are worth considering. Stainless steel orthodontic archwires could become magnetized, potentially affecting sliding friction, torque, and alignment (24), and patients with electronic implanted medical devices such as pacemakers should be monitored, since magnetic fields may interfere with device function (25, 26). We tested the archwire question directly: modelling the wire by reclassifying nearby mesh elements under a magnetic permeability representative of nickel-titanium or austenitic stainless steel archwire changed the inter-bracket field by less than 0.5% at any site, and even under a conservative worst-case permeability well above any wire alloy used clinically, most sites changed only modestly; the localized changes of up to 53% observed near the ends of the modelled wire span most likely reflect the finite (truncated) wire segment used in this arch model rather than a physical effect of a continuous clinical archwire, and would need re-examination with a complete wire geometry. For the clinically representative case, these results suggest the archwire does not meaningfully perturb the simulated field. The bonding system itself is a further point of interaction, given the proximity of the adhesive/cement layer to the embedded magnetic elements. This field magnitude — approximately 28–680 mT, extracted from the primary model solution, and best regarded as an estimate of the field magnitude the bonding layer is exposed to rather than a precision dosimetry calculation, since a dedicated adhesive-layer mesh region was not constructed — falls within the range examined in several recent *in vitro* studies of static magnetic field exposure on dental adhesives and glass ionomer cements. Salih Al Qassar et al. found that storing orthodontic adhesive in a static magnetic field of 22.5–100 mT altered its shear bond strength and adhesive remnant index (27). Al hauly et al. found that exposing uncured orthodontic adhesive to a 50 Hz magnetic field of 10–50 mT increased its degree of conversion, assessed by FTIR-ATR (28). A related study by the same research group reported that static magnetic field exposure (0.225 T) reduced microleakage beneath orthodontic bands cemented with both conventional and resin-modified glass ionomer cement (29). Alkhayat et al. reported changes in the mechanical properties of glass ionomer cement following static magnetic field storage at a comparable intensity (30), and a broader review by Al Qassar et al. summarizes the current evidence on magnetic fields and adhesive performance in orthodontics and dentistry (31). Separately, Garcia et al. and de Oliveira Teixeira et al. have shown that dental adhesives deliberately doped with magnetic (iron oxide) nanoparticles and manipulated with an external magnet can alter dentin bonding performance (32, 33); these studies address a mechanistically distinct scenario — engineered magneto-responsive adhesives rather than a conventional adhesive's passive response to an ambient field — but are noted here as related evidence that magnetic fields can influence adhesive/dentin bonding chemistry more broadly. Taken together, this literature suggests the bonding layer's exposure to the fields generated by this bracket system is a plausible, testable consideration for future experimental work, rather than a purely theoretical concern. Notably, this near-bracket zone where biologically-active field magnitudes are plausible is not a narrow theoretical window: it spans the same few-millimetre range as the adhesive/cement bonding layer itself, meaning the one location where the simulated field reliably reaches biologically-relevant magnitudes is also a site with an independent, directly testable literature on bonding material properties (27, 29–31, 33). Together, these findings identify two experimentally testable areas of potential interaction near the bracket — biological responses reported in cellular/osseous studies, and possible effects on the adjacent adhesive/cement system — though neither provides direct evidence of periodontal or clinical efficacy of the proposed bracket, and this study does not perform the direct periodontal or osseous validation that would be needed to establish either.

The amount of magnetic field exposure in the tissues adjacent to the magnets remains, more broadly, an issue for future study. While static magnetic fields differ greatly from ionizing radiation, their long-term effects in biological systems remain largely unknown, and we are unable to draw conclusions about these effects from the present study, since the response of biological tissue was not investigated; it would be of interest to determine whether a local magnetic field applied directly to the periodontal tissues induces an effective cellular response that facilitates orthodontic tooth movement.

This study is not without its limitations. It is only a computational simulation, using a simple model that did not account for the unique morphology of periodontal ligament and alveolar bone structures particular to each patient, and it relies on analytical estimation using idealized approximations for force calculation, which can be influenced by deviations from the real oral environment; direct experimental analysis of the biological effects of static magnetic fields on the periodontium has not yet been conducted and remains a speculative area of research. The periodontal ligament and alveolar bone are also not assigned distinct material (magnetic permeability) properties in the current model. We tested the sensitivity of the field prediction to this simplification directly, by re-solving the model with the entire non-magnetic domain assigned a realistic diamagnetic tissue permeability (μr ≈ 0.999991, typical of soft tissue and bone) instead of vacuum; the resulting field changed by less than 0.001% throughout the model, indicating that the magnetic-property difference between air and these tissues is negligible for the present magnetostatic calculation. This test does not, however, substitute for explicit periodontal ligament and alveolar bone geometry: because that geometry is not reconstructed in the current model, the reported field values should be read as spatial-attenuation estimates from the magnet surface rather than as tissue-resolved exposure at a known anatomical location, and this remains a limitation independent of the negligible permeability difference just described. This finding concerns the magnetic field prediction only, in any case: this study characterizes a magnetic field-generation platform, and — consistent with the force estimates above — does not model, and should not be interpreted as demonstrating, any biological or biomechanical effect of the generated field on the periodontal ligament, alveolar bone, or tooth movement. Obtaining true periodontal ligament and alveolar bone geometry would in any case require volumetric imaging (e.g., CBCT), which was outside the scope of the intra-oral surface scan used in this study.

A further limitation concerns mesh convergence. Table 3 demonstrates convergence of the peak flux density near the magnet across three mesh densities, but this near-field peak was the only quantity checked for mesh independence in the initial convergence study. This check was subsequently extended to the far-field/inter-bracket values that the biological comparisons above depend on, refining the mesh to 8× and 64× the original density (Table 4). The outer domain boundary treatment was found not to be a dominant source of error (a proxy test, since the original mesh geometry could not be regenerated with a larger air domain), but the far-field values themselves are still changing by up to 40% between the second and third refinement levels tested, confirming they had not converged at the mesh density used for the values reported in Table 2 and are not yet converged even at the finest density tested here. A related observation is that the near-magnet peak itself did not stabilize under the same uniform refinement, increasing from 0.72 T to 1.04 T to 1.12 T across the three levels, rather than converging as the medium-to-fine comparison in Table 3 suggested; this pattern is characteristic of an unresolved geometric singularity, most likely at a sharp magnet corner or edge in the idealized cuboidal geometry, where the field is mathematically unbounded and any finite mesh reports a resolution-dependent value rather than a true converged peak. This does not affect the medium/fine comparison in Table 3, which used a different, independently-generated mesh series over a smaller refinement range and does not itself establish convergence under the much larger uniform refinement applied here; it does mean that further uniform refinement beyond the densities used in this study would not be expected to yield a more accurate peak, and that any future refinement effort should be geometry-aware (e.g., locally smoothing or rounding sharp magnet corners) rather than purely uniform. The force and field magnitudes reported in this study should therefore be treated as provisional pending targeted refinement of the far-field air mesh, ideally using an adaptive remeshing approach applied to the original CAD geometry rather than uniform global refinement, which is nearing the practical limit of what this study's computational resources can support.

Further studies should aim to investigate the feasibility of prototyping the magnetic brackets and performing experimental studies in a laboratory setting; field mapping could be conducted to assess magnetic flux density under more realistic clinical conditions (34). It would also be useful to quantify any impact of an embedded magnetic element on the frictional coefficients of standard orthodontic archwires and subsequent orthodontic forces. Simulations should ideally incorporate models of varying occlusion types and different orthodontic bracket geometries, to better predict spatial magnetic flux distribution under different clinical scenarios, and careful attention will need to be paid to material selection for both the magnet and its coating, for corrosion resistance, durability, and biocompatibility, before *in-vivo* studies can be considered.

Despite these limitations, this investigation has confirmed that it is possible to create localized static magnetic fields by embedding tiny rare-earth magnets within a standard orthodontic bracket system, while maintaining a semblance of the current bracket design. The mechanical forces delivered are not powerful enough for effective orthodontic treatment; the system may instead have more promise as a method of delivering a localized static magnetic field capable of influencing the biology of tooth movement.

## Conclusion

This study demonstrated the feasibility of generating localized static magnetic fields by embedding miniature Nd–Fe–B magnetic components within orthodontic brackets. The calculated magnetic forces between adjacent brackets were substantially lower than conventional orthodontic force levels, suggesting that the proposed system may be more suitable as a localized static magnetic field delivery platform rather than as a direct force-generating appliance. Although such an approach may hold potential for influencing biological processes relevant to orthodontic tooth movement, no conclusions regarding its clinical efficacy can be drawn from the present findings. As this study was limited to computational simulation, further experimental validation and *in vitro*, *in vivo*, and clinical investigations are required to establish the biological effects and clinical relevance of the proposed system before any clinical application can be considered.

## Data Availability

The datasets presented in this study can be found in online repositories. The names of the repository/repositories and accession number(s) can be found below: Zenodo repository (doi: 10.5281/zenodo.20460602).
